# Supplementary material for: Are carbon emissions trading and green financial instruments synergistic? -Comprehensive quantitative research based on content analysis
Source: PLoS One. 2024 Mar 7;19(3):e0298601. doi: 10.1371/journal.pone.0298601 (PMC10919601; doi:10.1371/journal.pone.0298601)
Supplement: S1 Data — (ZIP) [file pone.0298601.s001.zip › Data/地级市Z_F工作报告.zip!/║┌┴·╜¡/╥┴┤║╩╨/└┤╫╘╣1⁄2╓┌║┼í╛┬φ┐╦╩2╛▌═°í┐_2018.docx]

各位代表：

现在，我代表市政府向大会作工作报告，请予审议，并请市政协委员和列席会议的同志提出意见。

一、2017年工作回顾

2017年是新一届政府履职施政的第一年。面对期望与重托，在市委的坚强领导下，在市人大、市政协的有力监督支持下，我们深入贯彻习近平总书记系列重要讲话特别是对我省两次重要讲话精神和对伊春的特指要求，按照省、市第十二次党代会的决策部署，团结带领全市人民，务实进取，攻坚克难，干事创业，圆满完成了市十四届人大一次会议确定的各项目标任务，全市经济社会发展取得较大进展。

——增长措施务实推动，经济运行稳中向好。面对经济下行压力，出台培育规上工业、降低企业成本等系列稳增长政策，采取月调度、季分析、大项目包保、一企一策等推动措施，着力解决制约经济发展的突出问题，主要经济指标全面完成年初既定目标。预计，全年地区生产总值实现266亿元，同比增长6.2%；规模以上工业增加值实现25亿元，增长13%；固定资产投资完成88.9亿元，增长17%；社会消费品零售总额实现131亿元，增长10%；公共财政预算收入完成15.7亿元，增长11.6%；城镇常住居民人均可支配收入达到23631元，增长6.5%；农村常住居民人均可支配收入达到13622元，增长6.2%；三次产业结构优化调整为42:20:38。全市经济彻底走出了全面停伐以来连续两年负增长的“低谷”，呈现稳中有进、进中向好的态势。

——资源优势初步释放，主导产业加快发展。扬绿水青山之长，借冰天雪地之势，着力挖掘经济增长点，推进五项主导产业向中高端迈进。森林食品业竞争力持续增强。蓝莓种植达6.95万亩，食用菌达5.6亿袋，森林猪、森林鸡、林下牛、森林雪貂饲养量分别达46.6万头、40万羽、10万头和17万只。全市各类种养基地达261个，“三品一标”作物达294万亩，新增绿色有机作物23.6万亩。忠芝集团研发有机蓝莓酒、木耳果汁等20余种新产品，预计实现产值1.1亿元。宝宇森林猪年屠宰1.2万头，加工香肠、肉罐制品120吨，产品销往哈尔滨、北京等地95家大型商超，预计实现产值2.6亿元。我市制定的“蓝莓酒”国家标准正式实施，“宝宇雪猪”获国家有机认证。伊林集团与汇源、佳龙集团合作建设的4个木耳菌包厂，4条果汁、果浆和矿泉水生产线，以及坚果、山野菜加工、坚果植物油萃取等项目相继建成投产，“伊林”品牌产品即将投入市场。森林生态旅游业大幅升温。全面推进全域旅游示范区建设, 森林康养、避暑休闲等主题产品市场份额扩大，九峰山养心谷、乌马河养溪谷、岐黄养老养生苑等一批新业态项目火热运营，桃山桃源湖旅游区被认定为国家4A级景区。成功举办了首届旅发大会，特色小镇、山庄建设全面启动，林都“十大最美景区”创新推出。冬季旅游市场破题搅热，滑雪、雾淞、温泉、狩猎等特色产品备受市场青睐，成功跻身中国冰雪旅游城市目的地排行榜前十位，荣膺“向全球游客推荐的生态旅游目的地”。“旅游+”战略有效实施，伊春国际森林马拉松等品牌赛事影响力扩大。预计全年接待游客1258万人次，旅游收入达113亿元，分别同比增长25.4%和29.6%。北药业稳步发展。南岔、汤旺河、红星、铁力局4个“森林百草园”加快建设，各类药材基地达97个、种改面积50万亩，红星平贝获批全省首个标准化药材种植示范基地。南岔格润药业甜叶菊提取、汤旺河天一药业苍术饮片加工等项目进展顺利，铁力葵花药业研发的国家级新药获临床批复，喜人药业升规入统。预计全年北药业实现产值20亿元，同比增长19.2%。木业加工业产销两旺。光明集团创新推出6大系列环保家居产品，主营业务增长3.4%，获批“第一批省级电子商务示范企业”。美江、永达等企业木艺产品年产量突破100万件，“北沉香”市场行情看好。预计全年木业加工业实现产值13.5亿元，同比增长3.1%。绿色矿业产能有效释放。鹿鸣钼矿生产钼精粉2.7万吨，预计全年实现销售收入13.6亿元。西钢集团稳定运行，预计实现销售收入75亿元。华能热电全面投用，预计实现销售收入9.6亿元。园区集聚效应初步显现，翠峦园区入驻各类企业85户，其中森林食品加工企业42户。铁力园区入驻企业30户。全市五项主导产业增加值占GDP比重达42%。

——管治并举全面保护，生态建设扎实推进。深入践行“生态就是资源，生态就是生产力”理念，严格实施天保二期工程。坚持“严管林”，查处各类涉林案件283起，依法恢复林地118公顷。科学经营森林，完成更新造林13.5万亩，抚育中幼林298万亩，森林蓄积年净增1000万立方米以上。林场（所）撤并稳步推进，乌马河紫貂自然保护区晋升为国家级自然保护区。森防建设不断加强，实现连续14年未发生重特大森林火灾。从严落实环保责任，科学划定生态红线，大力推行“河长制”，认真开展领导干部自然资源资产审计试点。狠抓环境治理，完成污染减排项目5个，累计淘汰建成区内小锅炉104台、黄标车及老旧车辆4859台，环境空气质量优良天数达94.2%，综合指数位列全省第一。

——改革攻坚不断深化，发展活力日益增强。严格落实中央6号和省委36号文件精神，制定了《伊春重点国有林区改革总体方案》及重点国有林管理分局组建等10个子方案。五营重点国有林管理局组建完毕，新青、铁力局改革试点及“南四局”企业办社会职能剥离稳步推进。政企合一区（局）公务员登记初步完成，事业单位离退休人员相应待遇得到落实。伊林、伊旅、供热三大集团组建完毕、投入运营。农村土地确权颁证工作基本完成。行政审批、财税金融体制改革统筹推进，铁力“四宝”在新三板成功挂牌，实现全市零的突破。对俄林业开发合作持续加强，赴俄采伐木材55万立方米，加工80万立方米，劳务输出2600人。对外交流稳步拓展，与芬兰凯米耶尔维市缔结国际友好城市，与广东茂名建立对口合作关系。预计全年引进外来投资项目160个，资金90亿元，同比增长21%。全市外贸进出口完成6310万美元，同比增长13.6%。

——基础设施逐步完善，城市建设成效显著。扎实推进国家新型城镇化综合试点，人居环境明显改观。2.7万户保障性安居工程全面开工，改造老旧小区35个，实施既有建筑节能改造16.5万平方米。“三供三治”加快建设，新建污水处理厂11个，供水、供热、供气普及率分别达88%、82%和85%。“大通道”建设加速推进，经努力争取，铁力至伊春铁路客运专线启动前期，林都机场改扩建工程顺利获批，伊汤公路及4条旅游公路加快建设，市公路客运总站开工建设。廻龙湾大街、供热一级网改造、锦绣山水公园等一批市政设施、绿化景观相继建成。“智慧伊春”时空信息云平台项目建成投用。经过全市上下九年的不懈努力，合力攻坚，成功摘得“第五届全国文明城市”桂冠。以创文为牵动，全市上下凝心聚力、锐意进取、埋头苦干的正能量加速汇聚，伊春知名度、美誉度和影响力日益攀升！

——民生实事有效落实，人民生活持续改善。全市民生投入占公共财政支出比例达75%，年初承诺的10件民生实事基本完成。脱贫攻坚战取得积极进展，全年投入产业扶贫资金1.35亿元，精准退出贫困人口717人，4个贫困村出列。就业质量不断改进，98%的停伐富余职工实现转岗就业，城镇新增就业2.15万人，完成计划的107.6%。“五项保险”基本实现法定人群全覆盖。各项社会事业加快发展，义务教育在全省率先整体实现县域均衡发展目标，高考实现12连增。职业教育基础设施不断完善，职业学院实验楼、综合服务中心和学生公寓建成投用。公立医院改革顺利实施，异地就医实现全国结算，市妇幼保健中心和市中心血站加快建设，医疗保障能力进一步加强。打造推出《映像·伊春》等文艺精品。群众体育工作全省评比名列第一。培育主营业务收入500万元以上科技型企业26户，获批国家知识产权试点城市。社会大局和谐稳定，安全生产、社会综治力度加大，再次捧回全国社会治安综合治理最高荣誉“长安杯”。伊春成为全省唯一同时拥有卫生城、文明城和平安城三大金字招牌的地级市，百姓的获得感、幸福感、安全感再度增强！

——作风建设深入开展，行政效能明显提高。政府系统“学讲话、转作风、优环境、争一流”活动务实推进。简政放权力度加大，取消、暂停和下放行政审批174项，颁发“多证合一”执照1600个，新增民营企业1810户。诚信建设切实加强，公布了“红黑名单”，查处各类案件40起。人大代表意见建议和政协委员提案回复率100%，群众反映强烈的突出问题得到较好解决，政府公信力、执行力有效提升。

各位代表，政府过去一年工作所取得的成效，主要得益于市委的统揽全局、坚强领导；得益于市人大、市政协的有效监督、大力支持；得益于全市各级干部和广大群众的担当尽责、辛苦付出。在此，我代表市政府，向全市人民及所有支持、参与伊春建设的各界人士，表示衷心的感谢和崇高的敬意！

回顾一年来的工作，我们也清醒地认识到，当前政府工作还存在不少问题和差距，还面临不少困难和挑战，主要是：经济总量小，发展不足；产业发展不充分，市场化程度低；深化改革任务艰巨，推进力度仍需加大；城市建设欠账多，基础设施还不完善；一些涉及百姓切身利益的问题尚未彻底解决，在发展中提高保障和改善民生水平还有许多工作要做；政府系统作风建设仍需深化，发展环境有待进一步改善。对此，我们将认真面对，努力加以解决。

二、2018年工作安排

2018年是贯彻党的十九大精神的开局之年，是决胜全面建成小康社会、实施“十三五”规划承上启下的关键一年。按照市委部署，明年政府工作的总体要求是：坚持以习近平新时代中国特色社会主义思想为指引，深入贯彻落实党的十九大精神、中央及省市委全会和经济工作会议精神，坚持稳中求进工作总基调，坚持新发展理念，紧扣社会主要矛盾变化，按照高质量发展的要求，统筹推进“五位一体”总体布局和协调推进“四个全面”战略布局，坚持以供给侧结构性改革为主线，统筹推进保生态、稳增长、调结构、促改革、惠民生等重点工作，坚决打好转方式调结构、产业项目建设、精准脱贫、国有林区改革、污染防治、防范化解重大风险、整顿作风优化发展环境攻坚战，坚定不移走绿色化转型发展之路，加快全面建成小康社会，加快建设富强民主文明和谐美丽现代化新伊春。

主要预期目标是：地区生产总值增长6.5%左右，固定资产投资增长10%，规模以上工业增加值增长7%，社会消费品零售总额增长10%，公共财政预算收入增长8%，城乡居民收入增长与经济增长同步。

为实现上述目标，突出抓好以下八项工作：

（一）严守红线不动摇，高标推进生态建设。坚持像对待生命一样对待生态环境，认真落实最严格的生态环境保护制度，努力打造好全国生态文明先行示范区。

全面加强生态保护。坚持保护优先、自然恢复为主，确保守住面积不减量、看住林木不盗伐、防住林火不出灾、治住病虫不出害、整治环境不污染。严格落实“严管林”各项措施，严厉打击各类破坏林木、林地及湿地行为。坚持“林区大事，防火第一”，加强森防能力建设，强化依法治火，确保不发生重特大森林火灾。加强自然保护区监管，积极推进“一区一法”建设。加快智能化大数据林业管理系统建设。探索启动红松国家公园试点建设，年内完成规划编制。推进第二轮森林资产及生态服务价值评价，提升生态系统的保障性和稳定性。

大力培育后备森林资源。注重提高森林质量，力促小兴安岭针阔混交林尽快恢复原貌。推进27个国营苗圃标准化建设，加快发展苗木花卉产业，力争实现产苗1亿株。强化造林管理，提高红松和“三大硬阔”营造比例，全年更新造林8万亩，完成中幼林抚育270万亩。强化林业有害生物监测，完成综合防治120万亩。发展碳汇经济，摸清碳汇底数，组建经营公司，统筹碳汇项目开发。

坚决打好污染防治攻坚战。严格落实生态环境损害责任追究办法，深入整改国家和省环保督察反馈问题，切实抓好生态红线划定工作。强化“河长制”宣传，抓实落靠各级主体责任，推进“一河一策”方案编制实施。严守水资源“三条红线”，推进河流治理、节水灌溉、饮用水源地污染防治等项目建设，强化畜禽养殖污染控制。严控建成区新建燃煤锅炉，淘汰10蒸吨以下燃煤锅炉，全面完成节能减排任务，打好蓝天保卫战，全年空气质量优良天数达95%以上，让伊春的绿水青山长留、蓝天白云永驻！

（二）深挖优势潜力，着力调整经济结构。坚持以供给侧结构性改革为主线，深入挖掘一二三产特色优势、供给潜力，找准突破口，抓住关键点，打好转方式调结构攻坚战。

做大一产抓融合。坚持把生产有机农产品作为种养业发展的终极目标，逐年加大推进力度，加快构建现代农业“三个体系”，促进一二三产融合发展。做大一产基础，着力调整种植结构，力争水稻、大豆、鲜食玉米、设施蔬菜种植面积分别达到68万亩、220万亩、4万亩和0.5万亩。加快建设绿色有机农产品种植基地，实施“三减”和增施有机肥，力争全市“三品一标”作物达到295万亩，新增绿色有机认证产品10个以上。提高农业生产机械化水平，培育壮大新型经营主体，新增农机专业合作社3个、生产经营型合作社5个、家庭农场20个。加强农田水利工程建设，扩大高效节水灌溉面积，力争新增3.9万亩。抓好生态畜牧业发展，推进铁力金新农和嘉荫林大牧业生态猪、宝宇森林猪、伊润种貂等项目建设，全市森林猪、森林鸡、林下牛、雪貂分别达到50万头、43万羽、10.8万头和18万只，人工繁育林蛙养殖面积达31万公顷。促进一产二产融合，以“粮头食尾”“农头工尾”为抓手，鼓励龙头企业深度开发“原字号”，推进宝宇森林猪肉制品、九河泉大米等项目建设，提高农副产品加工效益。促进一产三产融合，推动休闲农业发展，壮大山庄、沟谷经济，着力建设上甘岭溪水产业园、乌马河养溪谷等集田园采摘、休闲观光、饮食体验为一体的10个特色山庄。大力发展“互联网+农业”，构建线上线下营销体系，促进农业与旅游、物流等现代生产生活服务业有效融合。

做优二产抓提升。深入推进“三去一降一补”，重点在“破”“立”“降”上下功夫，加快提升工业质量和效益。提升重点骨干企业生产能力，保障生产要素，推动西钢、鹿鸣钼矿、华能热电、北方水泥等企业盘活存量、释放增量，力争优质钢材、钼精粉、水泥产能分别达到300万吨、2.8万吨和100万吨。提升规上企业总量规模，打好规上工业保卫战，注重对规上企业和临规企业的培育支持，进一步规范和取消涉企收费，降低融资成本，降低生产成本，使企业更具竞争力，力争净增规上企业10户以上。提升小微企业发展水平，出台支持政策，强化服务保障，常态化组织开展“银企对接”，扩大“助保贷”范围和信贷规模，力争新增小微企业230户以上。支持忠芝、越橘庄园等企业在“新三板”挂牌。充分运用“大智移云”互联网新技术，促进企业优化升级。

做活三产抓拓展。深入实施“旅游+”战略，推动电子商务、会展物流、健康养老等现代服务业加快发展。推动电商产业园、汇源物流园投入运营，力争分别引进企业60户、10户。

推动会展经济发展，举办好第五届中国（东北亚）森林博览会、中国金融40人伊春论坛等品牌展会活动。培育健康养老新业态，加大对岐黄养老养生苑、南岔永翠河国际健康疗养院、美溪廻龙湾全总职工疗养中心的营销力度，吸引更多群体入驻。推进金融业创新发展，促进信贷投放，力争全市各项贷款总额增长8.5%。推动文体产业发展，围绕建市60周年、开发建设70周年，组织开展文体主题活动，重点抓好森林音乐会等演艺精品，进一步提升国际森林马拉松等品牌赛事影响力。

（三）突出提质增效，着力发展绿色生态产业。牢固树立和践行“绿水青山就是金山银山，冰天雪地也是金山银山”的理念，以做好“三篇大文章”为主抓手，推动质量变革、效率变革、动力变革，大力发展实体经济，加快构建以绿色生态产业为支撑的现代林业经济新体系。

森林生态旅游业突出提档升级。坚持把森林生态旅游业作为第一引擎产业，以“山水田园综合体”理念为核心，加快创建国家全域旅游示范区，着力推动产品、营销、服务“三提升”。提高产品供给质量，继续打造康体养生、避暑度假、精品民宿、冰雪体验等新产品，加快推进汤旺河石林景区玻璃栈道、新青户外运动谷、带岭研学营地民宿客栈、南岔“红地盘”山洞游等项目建设，力争A级以上景区达到30个。围绕赏雾凇、泡温泉、滑雪狩猎等主题，打造更多参与性、体验性冬季旅游产品，举办好国际冬泳、林海雪原穿越等品牌赛事，全面叫响“林都雪城”品牌。强化宣传营销，推动旅游线路串联整合，与哈尔滨、五大连池等地共同开展区域营销。提高旅游服务能力，加快建设游客集散服务中心，完善公路重要节点旅游交通指示牌，在4A级以上景区实现WiFi全覆盖。推进国有景区公司化改制。推动伊旅集团全面运营，成立旅游联盟，注册客运公司，打造牵动全市旅游发展的市场载体。积极筹备承办第二届全省旅游产业发展大会。力争全年入境游客和旅游收入实现20%双增。

森林食品业突出扩量提质。坚持把森林食品和农林产品精深加工业作为第一支柱产业来打造，把产自小兴安岭的优质有机原料，加工出好产品，塑造出好品牌，卖出好价钱，加快建设“森林食品之都”。稳步扩大基地规模，推进菌包厂、有机黑木耳大棚，以及小浆果、山野菜种植栽培基地建设，新增木耳1亿袋，蓝莓、树莓、蓝靛果1.5万亩，山野菜2000亩，绿色速冻蔬菜3000亩。提高精深加工能力，重点推进小浆果、坚果、小食品、矿泉水、桦树汁饮品等26个项目加快建设。加强与东林、东农、哈工大等大专院校和科研院所合作，研发蓝莓花青素、黑木耳酵素等系列产品，推进中盟红松籽、黑尊黑木耳、铁力四宝林蛙、汤旺河九鑫山珍白酒等项目投产达效。深入实施品牌战略，推进“质量伊春”建设，放大伊春蓝莓、伊春黑木耳地理标志品牌效应，全面叫响“伊林”“小兴安岭大森林”绿色有机食品品牌。强化整体营销，在汇源物流园新建有机黑木耳大市场，做强“1林GO”电商平台，推进森林食品由种得好、养得好、采得好向卖得好转变。

北药业突出产业化发展。推广“基地+企业”合作模式，推动基地成规模、品种深加工，加快建设“林都北药之乡”。继续推进四个“森林百草园”建设，稳步扩大刺五加、返魂草、苍术等骨干品种种植规模，推广灵芝、黄芪等新品种种植，力争北药种改面积达到52万亩。围绕道地药材就地加工，积极招引大型药企落户伊春，推进格润药业植物提取、金北药口服液、天一药业饮片加工等项目加快投产，推进红星平贝梨汁项目扩大产能，形成更多拳头产品，提升林都北药影响力。

木业加工业突出品质提升。支持光明、双丰家具等龙头企业，提升智能制造水平，开展高档实木家具、家装定制业务，加快由机械化向自动化、智能化迈进。支持美江、永达等木艺企业提高创意水平，开发特色旅游纪念品，增强“北沉香”等木艺产品竞争力，加快建设“中国木艺之乡”。

绿色矿业突出增加效益。加快钼矿技术改造，提高生产加工能力，推进钼矿尾矿综合利用，力争实现销售收入15亿元、利税5亿元。支持西钢战略重组，调整产品结构，努力打造百亿元销售企业，力争实现利税8亿元。推进西林铅冶炼项目融资建设。推进高锶矿泉水以及桃山、乌马河等区域地热项目加快建设。加大“桃山玉”“北红玛瑙”推介力度，努力扩大市场份额。推进省地质勘查三年专项项目，挖掘矿业经济发展潜力。

（四）狠抓产业项目建设，加快培育经济增长点。深入实施产业项目建设三年攻坚战，坚持“全员抓招商、全力上项目”，保进度、促投产、求达效，切实激发项目牵动力。全年安排省市级重点产业项目40个，总投资131.5亿元，年度计划投资25.7亿元。

加强重大项目谋划储备。立足实际，整合资源，重点围绕绿色生态产业发展，谋划一批投资大、科技含量高、经济效益好、对地方经济有较强拉动力的大项目、好项目。全年谋划5000万元以上项目100个，其中超亿元重大项目40个。

务实推进项目招引落地。围绕三年招商引资指标任务精准对接，着力引进一批投资额大、带动力强、支撑性好的大项目。守住污染环境、破坏生态、高耗资源的项目坚决不上的底线。每个县（市）区（局）至少招引亿元以上项目1个。重点加强与汇源、佳龙、深圳九五、恒大农牧、华大基因等战略投资者的精准对接，促进意向项目尽早落地转化。支持本土企业加快成长，促进外商、内商共同发展。

加快项目建设进度。做好要素保障，抓好要件审批，力争汤旺河森林温泉度假酒店、铁力市黑臻黑木耳、乌马河颐养家园等10个新建项目按期开工，汇源绿色产业谷二期、伊春米兰小镇、带岭大青川石材开发等19个续建项目加快建设，确保按计划完成投资。

力促项目达产达效。从严落实落靠项目包保责任，注重提前介入，一企一策地解决水电热、资金、运输等生产瓶颈问题，促进现有项目快达产、见实效。重点推进新青、带岭黑木耳菌包厂，桃山森林冰雪玉温泉、朗乡冰雪花海航母、乌伊岭北红玛瑙玉石等11个项目竣工投产，形成更多新的经济增长动能。

增强园区承载能力。加快重点园区建设，强基础、引企业、建项目，切实发挥产业集聚功能。推进翠峦园区蒸汽管网建设，满足食品加工企业用汽需求，力争新引进企业10户左右、达产达效5户。推进铁力园区“一区两园”及污水处理、道路、燃气等设施建设，力争新引进企业10户左右、达产达效5户。

（五）聚焦重点难点问题，深化林区改革开放。以国有林区改革为统领，抓住重点，压茬推进各项改革任务，切实增强市场活力、社会创造力和开放竞争力。

加快推进国有林区改革。深入实施《伊春重点国有林区改革总体方案》，按照“四分开”时间节点要求，一季度完成伊春重点国有林管理机构组建并挂牌运行。完善伊林、伊旅、供热集团运营管理机制，推动伊林集团四大板块拓展运营，带动实体经济发展。加快在林管局、林业局两级组建培育实体公司。推进森工企业剥离社会职能，力争“南四局”企办教育顺利移交地方。全面完成公务员登记，清理规范各区局事业单位编制，适时启动“十定”工作。积极推进撤区设县、撤乡设镇工作。

推进重点领域改革。深化财政预算管理和税收制度改革。支持金融机构创新产品，加快组建小额贷款公司或融资性担保公司，提高金融服务实体经济效率和支持经济转型能力。防范政府债务风险和金融风险。深化商事制度改革，推进“多证合一”，强化企业信用监管，持续激发市场活力。推动公安体制改革取得实质性进展。扎实推进农村集体产权制度改革，全面完成11个试点村改革任务。

提升对外开发开放层级。积极沟通协调，继续推动嘉荫口岸恢复通关。加快推进跨境产业园建设。深化对俄木材采伐加工、农业、矿产开发合作，扩大对外贸易，力争进出口总额增长3%。扎实推进援疆工作。抓好与广东茂名对口城市合作，深化与芬兰、日、德等国际友好城市联系交流，积极扩大合作领域。

（六）深入实施“双创”战略，全面激发林区创新创业活力。坚持将创新作为引领发展的第一动力，将创业作为促进转型的重要支撑，让我们的城市发展更具活力、更有动力。

提高科技创新对经济发展的支撑力。重点实施市级以上科技项目20项。强化企业技术创新主体地位，鼓励中小企业围绕森林食品、北药等产业发展，研发新技术，开发新产品。支持林科院、农研中心等科研院所与企业深度合作，促进科技成果转化。推动省农科院小浆果研究所落户伊春。推动中科院长春分院在伊春建立工作站。扎实推进国家知识产权试点城市建设，强化知识产权创造、保护和运用。

大力支持民营经济发展。认真落实支持民营经济发展实施意见，鼓励民营企业扩大投资、科技创新、拓展融资。支持以发展绿色生态产业为主的龙头民营企业，通过技术升级、主业扩张、兼并重组加快发展。不断完善市场准入负面清单制度，引导民营企业依法进入竞争性经营行业和投资领域。加快构建“亲”“清”新型政商关系，让民营企业放心发展、放手发展、放开发展。激发和保护企业家精神，让民营企业家在政治上有地位、经济上有实力、社会上有荣誉。

推动更多社会主体投身创业。发扬林区三次创业精神，鼓励引导新一代林区人，以新的思维方式，勇于突破变革，激情干事创业。支持科技人员、林业富余职工、大学生、农民创新创业。强化职高、林校、职业学院等院校职业教育和技能培训，建设中小企业孵化园和民营经济创业园，完善翠峦、友好等地创业平台建设，打造更加广阔的创业空间和舞台，让更多想创业、会创业、能创业的青壮年劳动力和大学生留在家乡、建设家乡、发展家乡！

强化人才引进和培养。人才旺则伊春兴。坚持将人力资源作为第一资源，认真落实集聚鼓励人才创新创业实施意见，统筹抓好以科技领军、企业家和实用人才为重点的人才队伍建设，完善激励保障机制，吸引各方人才向林区流动。

（七）坚持城乡统筹推进，加速建设美丽伊春。立足推动区域协调发展，加快新型城镇化综合试点建设，补短板、求突破、促均衡，努力让城乡更加美丽、更加宜居、更加文明。

大力完善城市基础设施。重点推进铁路、公路、机场等重大交通设施项目建设，争取伊春至汤旺河公路完成建设，10月末通车。汤旺河至嘉荫公路开工建设、铁力至伊春铁路客运专线上半年全线开工、机场改扩建工程10月份完工。完成西岭至鹿鸣钼矿、五营至森林公园等4条旅游公路建设。开工建设总长45公里的带岭至丰林林场、南岔至仙翁山2条旅游公路。完成市公路客运总站及嘉荫、汤旺河、金山屯客运站建设，新建五营客运站。强化“三供三治”保障，提高供热、供水质量，给水分户改造完成3000户以上，中心城区管道燃气入户2000户。加快推进上甘岭、五营2个污水处理厂以及乌伊岭、红星、带岭、朗乡、浩良河镇5个垃圾处理厂建成投用，开工建设西林垃圾处理厂。推动中心城区净水厂投入试运行。实施保障性安居工程，改造棚户区6111户，完成既有建筑节能改造16万平方米，改造老旧小区16个。

推动老城区改造提升。推进伊翠铁路原线拆除，建设伊铁公园，为市民打造一个集自行车绿道、步道、广场和游乐于一体的城市主题公园、绿色休闲长廊。启动伊春河两岸景观工程建设。推进新兴东大街、青山东大街等10条道路新建改造，完善管线、路灯、人行道、绿化带等设施，让路灯亮起来、绿化美起来。加快特色小镇建设，坚持政府引导、市场化运作，重点推进汤旺河旅游小镇、嘉荫恐龙小镇、天场·童话朗乡小镇等10个试点建设。加快发展以休闲度假为主的高端旅游地产、康养地产，牵动全市房地产业拓展市场，带动城市品位进一步提升。

补齐乡村建设短板。深入实施乡村振兴战略，制订全市三年乡村振兴规划，促进城镇基础设施、公共服务向乡村、林场（所）延伸覆盖。加快改善交通环境，实施农村公路窄加宽62公里、路网改善41公里、安保工程48公里、建制村通硬化路53公里。深化美丽乡村建设，做好全市文明村镇创建工作，加强林场（所）街道、庭院绿化美化以及进出区（局）、场（所）2公里绿色长廊建设。加强城乡厕所改造，尤其是旅游景区、干道沿线厕所改造。推进民族特色村镇建设。大力发展乡村（林场所）经济，加快建设一批产业兴旺、功能完善、服务配套、具有田园社区风格的中心林场。

全面提高城市管理水平。高起点、高品质规划建设城市，推进中心城近期建设、城市修补等规划编制，抓好智慧城市、海绵城市及地下综合管廊建设。利用“智慧伊春”时空信息云平台开展城市规划、管理等应用开发。加快公交智能调度中心建设，更新电动公交车50台。深入开展市容环境综合治理，持续推进“六项整治”，加强弃管小区、集贸市场管理，常态化巩固文明城创建成果，确保卫生城顺利通过复审。

（八）回应美好生活需求，提高保障和改善民生水平。坚持以人民为中心的发展思想，秉持“尽力而为，量力而行”的原则，把有限财力更多地向民生领域倾斜，一件事情接着一件事情办，一年接着一年干，不断满足林区百姓日益增长的美好生活需要。

坚决打好脱贫攻坚战。主攻产业扶贫软肋，全力推进19个省级贫困村、5个市级贫困村光伏产业项目落地见效。落实扶贫小额贷款政策，引导贫困户以“带资入社”“委托经营”等方式创业。加快补齐“三保障”短板，全面完成建档立卡贫困户危房改造、饮水安全工程等任务，制定贫困家庭学生学前至高中阶段资助政策，让其更有尊严、更幸福地完成学业。推动贫困人口参加城乡居民医疗保险及大病保险实现全覆盖。注重扶贫同扶志、扶智相结合，让贫困户有愿望、有技能脱贫。确保全市11个贫困村出列、3085人脱贫。

加快发展社会事业。优先发展教育事业。办好幼儿教育，继续实施公办幼儿园建设奖励政策，提高普惠性幼儿园覆盖率。推动义务教育由均衡发展向优质化迈进。完成市级义务教育学习网络空间建设。提高普通高中教育教学质量，努力实现高考连增。加强职业教育，培养更多实用人才、本土人才。依托电视大学，办好社区教育。加快建设“健康伊春”。广泛开展全民健身运动，办好10项以上大型体育赛事。深化医疗卫生体制改革，探索公立医院激励机制。推进市中心血站、残疾人康复中心、市妇幼保健中心竣工投用，推进林业中心医院、市第一医院、第五医院改扩建。加强与省内知名医院合作，全面提高医疗技术水平。增强基层卫生服务能力。筹建市县两级全民健康信息平台。加快发展老龄服务事业，推动居家养老与社会养老有机结合。加强食品药品安全管控，让人民用的安全、吃的放心。全力抓好大学毕业生、富余林业职工等重点群体就业，全年实现城镇新增就业2万人，城镇登记失业率控制在4.5%以内。

提高社会保障水平。坚持兜底线、织密网、建机制，加快建设覆盖全民、城乡统筹、可持续的多层次社会保障体系。扩大养老保险覆盖范围，努力实现应保尽保，推进养老金按时足额发放。实施城乡居民医疗保险市级统筹，完善门诊慢性病、大病保险等制度，提高基本医疗保险水平。发挥失业保险促进就业作用，降低失业保险费率，扩大失业保险基金支出范围，落实失业保险技能提升补贴政策。积极开展工伤保险基金省级统筹，提高工伤保险服务水平。继续提高低保和特困人员救助标准，做好低保边缘困难群众救助工作，确保遇急有助、遇困有帮。

全面加强社会治理体系和能力建设。巩固“平安伊春”建设成果，严格落实安全生产责任制，深入开展重点行业领域安全生产大检查和专项整治，坚决杜绝重特大事故发生。重视信访工作，畅通群众利益诉求表达渠道，依法及时就地化解群众合理合法诉求。全力以赴做好维护稳定、治安防控、公共安全管理工作。推进公共安全视频监控“雪亮工程”建设。加强和改进新形势下民族宗教工作。探索开展社区、村民自治试点，推动社会治理重心向基层下移。

三、全面加强政府自身建设二号首长

打铁还须自身硬。新时代、新使命对政府自身建设提出新的更高要求。我们必须把全面从严治党要求落实到从严治政之中，切实增强“四个意识”，坚决维护以习近平同志为核心的党中央权威，持续提升执政能力和水平，努力建设让人民满意的法治政府、务实政府、廉洁政府和服务型政府。

深入推进依法行政。落实“深化依法治国实践”新要求，深入推进“法治伊春”建设，强化政府系统法治和规则意识。规范完善重大行政决策程序以及招投标、政府采购等制度。精简行政审批，推进“一号”申请、“一窗”受理和“一网”通办，探索推行“不见面”审批。全面推行并动态调整“三个清单”。大力优化发展环境，从严惩处破坏发展环境的行为，着力解决“门好进、脸好看，事还是难办”的问题。切实加强“诚信伊春”建设，坚决纠正失信行为。自觉接受人大依法监督、政协民主监督和社会公众监督，认真办理建议提案，及时将合理意见纳入政府决策，不断提升公众参与度和满意度。

持续促进作风转变。扎实开展“不忘初心、牢记使命”主题教育。倡导“五个好作风”，争做“三个好把式”，坚持干字当头，实字托底，铆紧各方责任，以钉钉子精神、工匠精神做实做细做好各项工作。创造性地贯彻新发展理念，不断提升专业素养，增强狠抓落实本领。健全完善目标考核体系，跟踪督办重大决策、重点工作，以激励促落实，以问责求实效，推动政府系统形成越是艰巨的任务越主动请战、越是困难的事情越要干出业绩的良好风气，切实汇聚起担当尽责、干事创业的正能量，扎扎实实地推动各项工作取得新成效。

自觉践行为民宗旨。牢记为人民谋幸福的使命要求，努力使各项决策更近民情、更顺民意、更贴民心。凡是对人民作出的承诺，都要言必行、行必果、果必真。切实把人民群众所盼的，变成政府真干的；切实把政府所干的，变成人民群众真盼的。坚持深入基层、深入群众，多渠道、接地气的了解社情民意，精准破解群众反映强烈的困难和问题。扎实开展行风测评、公开述职评议等活动，“听民之所呼、问民之所需”。扩大政务公开范围，提高政府工作透明度，加快建设“互联网+政务服务”平台，让信息、数据多跑路，让企业、群众少跑腿。

切实强化廉政建设。坚决执行中央八项规定和省市相关规定，严格落实“一岗双责”，持续推进正风肃纪，让干部知敬畏、存戒惧、受监督。加强对公共资金、国有资产、国有资源等领域的审计，严控岗位廉政风险。继续压缩行政运行成本，确保“三公”经费“零增长”。驰而不息纠正“四风”，规范权力运行，重拳查处侵害群众利益的不正之风和腐败问题，营造廉洁奉公、风清气正的政治生态环境。

各位代表！新时代赋予新使命，新时代要有新作为。让我们更加紧密地团结在以习近平同志为核心的党中央周围，在市委的坚强领导下，不忘初心、牢记使命，以永不懈怠的精神状态和一往无前的奋斗姿态，苦干实干创新业，砥砺奋进踏新程，全力推动伊春绿色化转型发展，让老林区焕发青春活力，努力谱写建设富强民主文明和谐美丽现代化新伊春的宏伟篇章！
